# Association of serum cholesterol with Parkinson's disease in a cohort of statin‐free individuals

**DOI:** 10.1002/brb3.2454

**Published:** 2021-12-11

**Authors:** Yukai Lv, Bo Xu, Xuejuan Zhang, Chunhuan Chen, Yan Gao, Ning Li

**Affiliations:** ^1^ Department of Pediatrics The First Affiliated Hospital, Sun Yat‐sen University Guangzhou China; ^2^ Department of Thoracic Surgery The First Affiliated Hospital, Sun Yat‐sen University Guangzhou China; ^3^ Department of Radiology The First Affiliated Hospital, Sun Yat‐sen University Guangzhou China; ^4^ Department of Anaesthesiology Jinshan Hospital, Fudan University Shanghai China; ^5^ Department of Neurology Affiliated Hospital of Hebei University Baoding China

**Keywords:** association, low‐density lipoprotein statin, Parkinson's disease, serum cholesterol, statin

## Abstract

**Introduction:**

The role of serum cholesterol in the pathogenesis of Parkinson's disease (PD) remains unclear. The objective of this study was to assess the association between serum cholesterol and PD in a cohort of statin‐free newly diagnosed PD patients.

**Methods:**

This retrospective study used fasting lipid profiles obtained from 672 consecutive statin‐free newly diagnosed PD individuals and 540 controls. These PD individuals were identified from three medical institutions during 2017–2021, and the controls were identified from three physical examination centers during the same time period. Logistic regressions were used to estimate odds ratios (ORs) and 95% confidence intervals (CIs), with adjustment of age, sex, and tobacco use history.

**Results:**

Among 672 PD individuals, 112 were excluded in accordance with the current criteria, leaving 560 PD patients. The multivariate binary logistic regression analysis showed that LDL‐C was the only variable contributing to the occurrence of PD (OR 1.39, 95% CI: 1.07‐2.31, *p* < .001) after adjusting for age, sex, and tobacco use history; this association persisted following further adjustment for TC and HDL‐C. In the subgroup analysis of the adjusted results of LDL‐C after correcting for TC and HDL‐C, lower LDL‐C was associated with a higher risk of PD.

**Conclusion:**

Among selected populations of statin‐free newly diagnosed PD individuals, low LDL‐C might be associated with the occurrence of PD.

## Graphical abstract

Epidemiological evidence on PD with respect to serum cholesterol has not been well delineated. Among selected populations of statin‐free, newly diagnosed PD individuals, low LDL‐C might be associated with the occurrence of PD.

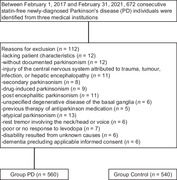

## 1 INTRODUCTION

Parkinson's disease (PD), a chronic, progressive neurodegenerative disorder characterized clinically by irreversible, progressive motor and nonmotor dysfunction, occurs mostly in the elderly (Jankovic, 2008; Uchida et al., 2020; Zarkali et al., 2021). Existing management decisions only temporarily lessen PD symptoms and fail to halt disease progression (Raza et al., 2019). A better understanding of the mechanisms and risk factors related to PD could improve disease management and help with elucidating causal pathways, as well as uncovering positive and negative correlations with other diseases (Aarsland et al., 2012). The mechanisms of neuronal death in PD are not fully understood, which makes exploring risk factors for PD challenging (Venkatesan et al., 2021; Wu et al., 2021). Furthermore, the nimiety of identified risk factors for developing PD makes it difficult to predict disease occurrence and development or to reflect its severity (Allam et al., 2003; Sawada et al., 2007). The progressive degeneration and loss of dopaminergic neurons from the nigrostriatal pathway, the formation of Lewy bodies, and microgliosis may be crucial pathological changes in PD, which result in a remarkable decrease in dopamine levels in the striatum (Oliveira et al., 2021; Ye et al., 2021). The particular etiology associated with the interplay among ageing, genetic susceptibility, and environmental factors remains unknown, although the identification of novel predictors of PD is becoming a growing area of interest (Latourelle et al., 2017; Macleod & Counsell, 2016). Ever‐increasing evidence (Trupp et al., 2014; van Wamelen et al., 2021) supports a multifaceted interaction between genetic, biological, and molecular abnormalities. The etiologic and physiological factors that contribute to this identification of prediction in the evolution of PD have become the focus of PD. Serum cholesterol, ageing, genetic factors, and oxidative stress may be associated with the degeneration and loss of dopaminergic neurons (Gudala et al., 2013).

Epidemiological evidence on PD with respect to serum cholesterol has not been well delineated (Gudala et al., 2013; Hu, 2010). Additionally, previous studies (Deischinger et al., 2021; Gudala et al., 2013) have not directly related the potential role of serum cholesterol to the etiology of PD, as the factors regulating serum cholesterol have been less extensively studied. To date, however, whether baseline serum cholesterol levels have a potential relationship with PD in a cohort of statin‐free individuals remains unclear. Little published literature on the role of serum cholesterol in the occurrence of PD has been reported. Hence, we launched a multicenter retrospective study to evaluate the association of serum cholesterol with the occurrence of PD in a cohort of statin‐free, newly diagnosed individuals.

## 2 MATERIALS AND METHODS

### 2.1 Data

Between February 1, 2017, and February 28, 2021, individuals with statin‐free, newly diagnosed PD for whom baseline data were available were identified from three medical institutions and were retrospectively analyzed. Inclusion criteria comprised the following: individuals aged ≥45 years at diagnosis, but aged < 80 years because PD individuals aged ≥ 80 years may result in an increased selection bias attributed to potential comorbidities; the clinical diagnosis of PD was consistent with previous recommendations of the International Parkinson and Movement Disorder Society criteria for PD (Postuma et al., 2018); individuals were free of acute medical events and key neurological disease (excluding PD) and had no prior use of statins; newly diagnosed PD individuals were eligible for this study. All methods were carried out according to the relevant guidelines and regulations of the institutional and/or national research committee and the 1964 Helsinki declaration. This study was strictly retrospective and involving the collection of existing data and records, and therefore, the need for informed consent was waived by the Institutional Review Board of the Affiliated Hospital of Hebei University. This study was approved by the board of the Affiliated Hospital of Hebei University (No. 16/3419).

The key exclusion criteria comprised a lack of patient characteristics; no documented parkinsonism; injury of the central nervous system attributed to trauma, tumor, infection, or hepatic encephalopathy; secondary parkinsonism; drug‐induced parkinsonism; postencephalitic parkinsonism; unspecified degenerative disease of the basal ganglia; previous therapy with antiparkinson medication (i.e., levodopa and carbidopa) to control motor symptoms or ameliorate the underlying disease; atypical parkinsonism; resting tremor involving the neck/head or voice; poor or no response to levodopa; disability resulting from unknown causes; and dementia precluding applicable informed consent. For the control group, individuals who were free of PD were identified from three physical examination centers that checked whether the patients in the control group had neurological disorders during the same time period.

The primary outcomes were serum cholesterol, including low‐ or high‐density lipoprotein cholesterol (LDL‐C or HDL‐C, respectively), and total cholesterol (TC). For individuals with newly diagnosed PD, a fasting lipid profile (LDL‐C, HDL‐C, and TC) was measured in the medical institution laboratory when the individual was admitted to the hospital. For the control group, the fasting lipid profile was determined from three physical examination centers in each medical institution. Baseline data (age, sex, tobacco use history) were available for all participants. For PD patients, blood samples were collected when PD was newly diagnosed. For control patients, blood samples were collected during physical examination. The estimation of PD duration was calculated from the PD diagnosis date during fasting lipid testing. Clinical history and laboratory data were collected by neurologists (YG and NL) using standardized forms.

### 2.2 Statistical analysis

The distribution of baseline data was extracted from administrative records. The primary comparisons involved the outcomes of LDL‐C, HDL‐C, and TC tested in the PD group versus the outcomes tested in the control group. A comparison of continuous variables at baseline was executed using Student's *t*‐tests for normally distributed variables and the Mann–Whitney U test for nonnormally distributed variables. Comparisons of categorical variables at baseline were executed using the chi‐square test or Fisher's exact test. Multivariate binary logistic regression was used to estimate the odds ratios (ORs) and 95% confidence intervals (CIs) for LDL‐C, HDL‐C, and TC to assess the association between serum cholesterol levels and the risk of PD, adjusting for age (by 5‐year groups from age ≥45 to < 80), sex, education, tobacco use history (never, current, and past), body mass index (BMI), and disease duration. The subgroup analysis on LDL‐C was conducted using quartiles. All statistical analyses were performed using the Statistical Package for the Social Sciences version 26 (SPSS Inc., Chicago, Illinois). A *p*‐value <.05 was considered significant.

## 3 RESULTS

### 3.1 Demographic characteristics

**FIGURE 1:**
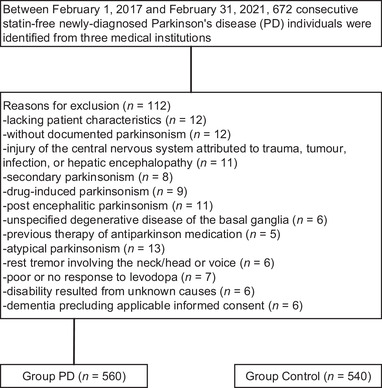
Flow diagram exhibiting methods for identification of participators to assess the association of serum cholesterol with Parkinson's disease (PD) in a cohort of statin‐free newly diagnosed PD individuals, and reasons for exclusion

We identified 672 consecutive statin‐free, newly diagnosed PD individuals, of whom 112 were excluded according to the established inclusion/exclusion criteria, as shown in Figure 1. Therefore, 560 individuals were included for analysis. Five hundred forty healthy controls were available. Table 1 summarizes baseline data for the individuals. The mean age was 69.3 years (range, 58–80) in the PD group and 68.7 years (57–80) in the control group. In the PD group, 52.0% of individuals (*n* = 291) were male, and 48.0% (*n* = 269) were female. In the control group, 53.3% of individuals (*n* = 288) were male, and 46.7% (*n* = 252) were female. Tobacco use history was never in 13.4%, current in 68.6%, and past in 18.0% of individuals in the PD group versus never in 21.7%, current in 38.3%, past in 40.0% of individuals in the control group (*p *< .001). At the time of serum cholesterol measurement, mean HDL‐C was 57.7(19.3) mg/dL in the PD group versus 57.2(21.6) mg/dL in the control group (*p *= .072). The baseline data were similar between the two groups, irrespective of disease duration, TC, or LDL‐C. Mean TC was 203.1(45.6) mg/dL in the PD group versus 220.5(54.5) mg/dL in the control group (*p *< .001). Mean LDL‐C was 124.6(41.6) mg/dL in the PD group versus 101.1(46.5) mg/dL in the control group (*p *< .001). No cholesterol‐lowering drugs were used in either group.

**TABLE 1:** Baseline data of participators included in the study

Factors	PD (*n* = 560)	Controls (*n* = 540)	*p*‐Value
Age*, years	69.3 (11.2)	68.7 (11.3)	.126^a^
Sex, n (%)			.650^b^
Male	291 (52.0)	288 (53.3)	
Female	269 (48.0)	252 (46.7)	
Education, years	14.8 (7.6)	15.2 (7.9)	.114^a^
Tobacco use history, *n* (%)			<.001^b^
Never	75 (13.4)	117 (21.7)	
Current	384 (68.6)	207 (38.3)	
Past	101 (18.0)	216 (40.0)	
BMI, kg/m^2^	25.7 (9.4)	25.1 (10.5)	.304^a^
Disease duration**, years	2.4 (1.1)		
TC, mg/dL	203.1 (45.6)	220.5 (54.5)	<.001^a^
HDL‐C, mg/dL	57.7 (19.3)	57.2 (21.6)	.072^a^
LDL‐C, mg/dL	124.6 (41.6)	101.1 (46.5)	<.001^a^

Continuous and categorical variables were represented as means (standard deviations) and numbers (proportions), respectively.

Abbreviations: PD, Parkinson's disease; BMI, body mass index; TC, total cholesterol; HDL‐C, high‐density lipoprotein cholesterol; LDL‐C, low‐density lipoprotein cholesterol.

^a^ Analyzed using the Student's *t*‐test for normally distributed variables and the Mann–Whitney U test for nonnormally distributed variables.

^b^ Analyzed using the chi‐square test or Fisher's exact test.

* Calculated based at time of fasting lipid profile testing.

** Calculated based at time of fasting lipid profile testing from the date of PD diagnosis.

Table 2 shows the multivariate binary logistic analysis of factors associated with PD in a cohort of individuals with newly diagnosed PD. Age, education, and tobacco use were not involved since these variables were considered independent factors for predicting the occurrence of PD. BMI, TC, and HDL‐C were not remarkable predictors (OR 1.01, 95% CI: 0.12–1.75; OR 1.18, 95% CI: 0.46–2.28; OR 1.22, 95% CI: 0.15–2.79, respectively). The multivariate binary logistic regression analysis showed that LDL‐C was the only variable contributing to the occurrence of PD (OR 1.39, 95% CI: 1.07–2.31, *p* < .001) after adjusting for age, sex, and tobacco use history and persisted following further adjustment for TC and HDL‐C. Table 3 shows the multivariate binary logistic regression model for the adjusted results of LDL‐C in a cohort of individuals with newly diagnosed PD. In the subgroup analysis of the adjusted results of LDL‐C after correcting for TC and HDL‐C, low LDL‐C was associated with a higher risk of PD. LDL‐C was 83–90 mg/dL (OR 1.35, *p* < .001) and 90–110 mg/dL (OR 1.78, *p* < .001) after adjusting for age, sex, and tobacco use history.

**TABLE 2:** Multivariate binary logistic regression analysis of factors associated with PD in a cohort of individuals with newly diagnosed PD

Factors	β	SE	OR**	95% CI	χ^２^	*p‐*Value
BMI	1.023	0.254	1.01	0.12–1.75	3.28	.265
TC	1.537	0.171	1.18	0.46–2.28	6.46	.104
HDL‐C	1.294	0.129	1.22	0.15–2.79	1.56	.077
LDL‐C	1.478	0.317	1.39	1.07–2.31	2.42	< .001*

Abbreviations: BMI, body mass index; TC, total cholesterol; HDL‐C, high‐density lipoprotein cholesterol; LDL‐C, low‐density lipoprotein cholesterol; SE, standard error; OR, odds ratio; CI, confidence interval.

* Statistically significant.

** Adjusted for age, sex, and tobacco use history.

**TABLE 3:** Multivariate binary logistic regression model for LDL‐C subgroup analysis in a cohort of individuals with newly diagnosed PD

LDL‐C (mg/dL)	β	SE	OR**	95% CI	χ^２^	*p‐*Value
83–90	1.380	0.126	1.35	1.15–2.79	3.92	<.001*
90–110	1.638	0.546	1.78	1.22–2.61	2.36	<.001*
110–130	1.341	0.427	1.63	0.91–1.97	1.42	.372
130–150	1.275	0.362	1.97	0.27–1.17	2.74	.259
150–165	1.549	0.345	1.01	0.38–3.40	2.01	.136

Abbreviations: LDL‐C, low‐density lipoprotein cholesterol; SE, standard error; OR, odds ratio; CI, confidence interval.

* Statistically significant.

** Adjusted for age, sex, and tobacco use history.

## 4 DISCUSSION

This retrospective study provides evidence that low LDL‐C may be associated with a high occurrence of PD in a cohort of statin‐free newly diagnosed individuals. As with previous studies (Huang et al., 2007; Kreisler et al., 2007), the current findings are constrained by the retrospective design, and causal inferences are lacking. The low incidence of PD in the population makes it challenging to initiate prospective trials to assess the relationship between cholesterol levels and PD. The sample size of this study was relatively small, which led to limited statistical capacity. However, this study may be the largest retrospective study to assess the association of serum cholesterol with PD in a cohort of statin‐free individuals.

A growing but still particularly inadequate body of literature (de Lau et al., 2006) has assessed the association of serum cholesterol with PD and indicated that lower serum cholesterol attributed to LDL‐C may be associated with a higher risk of PD. To date, published studies (Bao et al., 2018; Saedi et al., 2021) assessing the association of LDL‐C with PD in a cohort of statin‐free individuals remain limited and debated. Consistent with the current findings, Huang et al. (Huang et al., 2007) showed that low LDL‐C may be associated with the occurrence of PD and with age‐dependent changes in cognitive function. Similarly, Sterling et al. (Sterling et al., 2016) assessed the association between baseline LDL‐C and prospective changes in PD and showed that higher LDL‐C may result in improved fine motor function. Identifiable cholesterol‐cognitive relationships could be propelled by low LDL‐C associated with PD (Garcia‐Sanz et al., 2021). However, previous evidence (Rozani et al., 2018) also favors an association between high serum cholesterol and low PD occurrence, implying a favorable role of high serum cholesterol in delaying the occurrence of PD. Based on analogous premises, the results from DATATOP (Huang et al., 2011) showed that low LDL‐C may be associated with the progression of PD. However, the results from several studies (Garcia‐Sanz et al., 2021; Park et al., 2021; Yu et al., 2020) reported nonassociation between baseline LDL‐C and PD, which is inconsistent with our findings. Previous varied results may be attributed to distinctions in baseline data and cholesterol‐lowering medication use (Alfradique‐Dunham et al., 2021; Deischinger et al., 2021; Gudala et al., 2013; Jankovic, 2008). In addition, serum cholesterol levels do not represent cholesterol levels in tissues or cells (Rozani et al., 2018).

Although serum cholesterol may have a certain influence on the occurrence and development of PD, low LDL‐C may only be a trigger of PD (Gudala et al., 2013; Saedi et al., 2021). Hence, understanding the potential association between low LDL‐C and PD may be more clinically valuable. The pathogenic mechanism of PD may be associated with the abnormal interaction of cholesterol with α‐synuclein, instigating its detrimental aggregation and resulting in the loss of dopaminergic neurons (Garcia‐Sanz et al., 2021; Jankovic, 2008). Previous studies (Garcia‐Sanz et al., 2021; Gudala et al., 2013) have shown that intracellular cholesterol has a dual role of not only resisting lysosomal membrane permeabilization but also aggravating the accumulation of α‐synuclein. The process of neurotransmitter release is regulated by α‐synuclein (Garcia‐Sanz et al., 2021). Accumulation of α‐synuclein eventually leads to obstruction of neurotransmitter release (Gudala et al., 2013; Rozani et al., 2018). Likewise, the apolipoproteins (Apos) synthesized by astrocytes exist on brain cells and have three forms (ApoE, ApoJ, and ApoA1)(Garcia‐Sanz et al., 2021). The ApoE‐cholesterol complex is internalized into neurons mediated by LDL‐C receptors and LDL‐C receptor‐related protein (Garcia‐Sanz et al., 2021; Saedi et al., 2021). The increase in LDL‐C may initiate disturbances in cholesterol homeostasis, which may result in damage to neuronal cell membrane structure and loss of synapse function, and negative feedback from this process and may produce a detrimental vicious cycle (Garcia‐Sanz et al., 2021; Uchida et al., 2020).

Although results from previous reviews (Rozani et al., 2018; Saedi et al., 2021) have indicated a potential role of serum cholesterol in PD pathogenesis, the process of cholesterol metabolism is dynamic, accompanied by the formation of a large number of metabolites, and it is possible that one or more metabolites can lead to the occurrence of PD under specific conditions (Hu, 2010; Huang et al., 2007), which can well explain the sporadic epidemiology of PD. Based on this premise, it may be difficult to reach a consensus on the understanding of PD risk factors when analyzing only the pathological mechanism of PD (Huang et al., 2008). A meta‐analysis of four case‐control and cohort studies (Gudala et al., 2013) comprising 246,112 subjects assessed the association between serum cholesterol and PD and showed no association between serum cholesterol and the risk of PD, although several mechanisms favor the hypothesis of a heightened risk of PD in individuals diagnosed with excessive serum cholesterol levels. Nonetheless, the definitive relationship between serum cholesterol and PD may have been masked by a variety of confounding factors (Garcia‐Sanz et al., 2021). In this referenced meta‐analysis, newly diagnosed PD was not included, which may have contributed to the reported nonassociation.

Although the defined biological mechanisms of the serum cholesterol‐PD relationship remain indeterminate (Garcia‐Sanz et al., 2021), we hypothesize that cholesterol may promote the re‐repair mechanism of damaged PD‐related neurons. Cholesterol is the raw material needed to trigger synapses, and antagonizing LDL‐C receptors will block this biological process (Deischinger et al., 2021; Garcia‐Sanz et al., 2021). Nevertheless, in undamaged PD cells, cholesterol is synthesized predominantly by astrocytes and is then transported to neurons through LDL‐C receptors and ApoE (Garcia‐Sanz et al., 2021; Gudala et al., 2013). Given the restricted ability of serum cholesterol to infiltrate the blood‐brain barrier, brain cholesterol is produced predominantly de novo and is not directly associated with serum cholesterol levels (Deischinger et al., 2021; Gudala et al., 2013; Latourelle et al., 2017).

Several leading limitations should be emphasized. The retrospective design of the review and the subjects included in the study may have an effect on our results to a degree and prevent us from deducing any underlying association between serum cholesterol and the risk of PD. Additionally, the testing time of the serum cholesterol levels is variable, which may cause fluctuations in the detected values and the lipid measurements by different laboratories might impact the results. Furthermore, standardized criteria for diagnosing concomitance with diverse diseases are lacking in this study. Together with the participant's individual habits (i.e., dietary preferences, exercise volume, use of antihypertensive drugs, etc.), these data were collected by subjective oral recall of the participants and diabetes associated with both PD and high lipid levels were not involved in this study, which may have had some effect on the results. The generalizability of these results is also limited since this study was limited to statin‐free individuals aged ≥45 years at diagnosis.

## 5 CONCLUSION

The findings from the study may add to an increasing body of evidence that among selected populations of statin‐free PD individuals, lower LDL‐C tends to be associated with a higher risk of PD. Although the serum cholesterol‐PD relationship remains under dispute, the relationship may be driven by multiple risk factors associated with PD. The reduction in serum cholesterol may be only an early warning, which may result from the disorder of brain cholesterol homeostasis. Even if the association between serum cholesterol and PD is causal, considering the adverse effects of LDL‐C on cardiovascular and cerebrovascular diseases, establishing an applicable cholesterol target may be necessary. Future trials are merited to further validate these findings and explore the underlying mechanisms.

## CONFLICT OF INTEREST

The authors declare no conflict of interest.

## AUTHOR CONTRIBUTIONS

YG participated in the data collection and prepared the manuscript. BX and PW participated in the statistical analysis and the preparation of study protocols. NL provided guidance in analyzing the dataset and contributed to writing the manuscript. All authors read and approved the manuscript.

### PEER REVIEW

The peer review history for this article is available at https://publons.com/publon/10.1002/brb3.2454

## Data Availability

The data used to support the findings of this study are available from the corresponding author upon request.
